# Screening for Rare Mitochondrial Genome Variants Reveals a Potentially Novel Association between *MT-CO1* and *MT-TL2* Genes and Diabetes Phenotype

**DOI:** 10.3390/ijms25042438

**Published:** 2024-02-19

**Authors:** Tomasz Płoszaj, Sebastian Skoczylas, Karolina Gadzalska, Paulina Jakiel, Ewa Juścińska, Monika Gorządek, Agnieszka Robaszkiewicz, Maciej Borowiec, Agnieszka Zmysłowska

**Affiliations:** 1 Department of Clinical Genetics, Medical University of Lodz, Pomorska 251, 92-213 Lodz, Poland; sebastian.skoczylas@umed.lodz.pl (S.S.); karolina.antosik@umed.lodz.pl (K.G.); paulina.mludzik@umed.lodz.pl (P.J.); ewa.juscinska@umed.lodz.pl (E.J.); monika.gorzadek@umed.lodz.pl (M.G.); maciej.borowiec@umed.lodz.pl (M.B.); agnieszka.zmyslowska@umed.lodz.pl (A.Z.); 2 Department of General Biophysics, Institute of Biophysics, University of Lodz, Pomorska 141/143, 90-236 Lodz, Poland; agnieszka.robaszkiewicz@biol.uni.lodz.pl

**Keywords:** mtDNA, heteroplasmic, diabetes, pathogenic

## Abstract

Variations in several nuclear genes predisposing humans to the development of MODY diabetes have been very well characterized by modern genetic diagnostics. However, recent reports indicate that variants in the mtDNA genome may also be associated with the diabetic phenotype. As relatively little research has addressed the entire mitochondrial genome in this regard, the aim of the present study is to evaluate the genetic variations present in mtDNA among individuals susceptible to MODY diabetes. In total, 193 patients with a MODY phenotype were tested with a custom panel with mtDNA enrichment. Heteroplasmic variants were selected for further analysis via further sequencing based on long-range PCR to evaluate the potential contribution of frequent NUMTs (acronym for nuclear mitochondrial DNA) insertions. Twelve extremely rare variants with a potential damaging character were selected, three of which were likely to be the result of NUMTs from the nuclear genome. The variant m.3243A>G in *MT-TL1* was responsible for 3.5% of MODY cases in our study group. In addition, a novel, rare, and possibly pathogenic leucine variant m.12278T>C was found in *MT-TL2*. Our findings also found the *MT-CO1* gene to be over-represented in the study group, with a clear phenotype–genotype correlation observed in one family. Our data suggest that heteroplasmic variants in *MT-COI* and *MT-TL2* genes may play a role in the pathophysiology of glucose metabolism in humans.

## 1. Introduction

Disorders in glucose metabolism are very common among modern human populations, with genetic background being one of the primary causes. Indeed, modern genetic diagnostics have identified a number of variants in the nuclear genome, particularly in *GCK*, *HNF1B*, *HNF1A* and *HNF4A*, that appear to increase the risk of the development of maturity-onset diabetes of the young (MODY) [1]. Research also indicates that the onset of MODY may be hastened by heteroplasmic variants occurring in the mitochondrial genome. The best described heteroplasmic variant occurs in the tRNA gene for leucine at site m.3243A>G, known to cause maternally inherited diabetes and deafness (MIDD) syndrome [2]. However, recent reports indicate that other variants in the mtDNA genome may also be associated with diabetic phenotype [3,4].

A routine genetic diagnosis of monogenic diabetes does not typically include an analysis of the mitochondrial genome. Also, most such genetic variation is heteroplasmic, and any analysis therefore requires high genome coverage and a different methodological approach to data processing and interpretation. The following work is the only available attempt to screen the entire mitochondrial genome in such a large group of MIDD/MODY-eligible diabetic subjects. So far, only isolated cases have been subjected to such thorough analysis [4]. The main aim of this work is to identify key genetic variations in the whole mitochondrial genome in a group of individuals eligible for MODY/MIDD diabetes.

## 2. Results and Discussion

In 193 patients, a total of 901 unique mutations were identified in the mitochondrial genome with an average coverage of 1872× (Tables S2, S3, S6 and Figure S5). Twelve extremely rare variants with a potentially damaging character were selected; these are summarized in Table 1. The only variant that occurred in more than one patient was the m.3243A>G variant in the tRNA gene for the amino acid leucine. Three potential damaging variants in two patients that were likely to be the result of NUMTs from the nuclear genome are summarized in Table S4. Fourteen distinct major mtDNA haplogroups were identified in the study group. The exact list of haplogroups is included in Table S1.

### 2.1. tRNA Mutations

#### 2.1.1. m.3243A>G Variant

As expected, the most common variant was in the gene for leucine tRNA (*MT-TL1*) at site m.3243A>G. The variant was found in seven patients, which represented a 3.5% proportion of the study group. A number of reports have implicated the variant in the occurrence of the MIDD phenotype [2]. A segregation analysis performed for three families (Figures S1–S3) found the trait to be inherited via the maternal line. In one case, the variant appeared de novo in the patient. Almost all patients, both probands and their mothers, were distinguished by a diabetic phenotype and significant hearing loss, except for one case where no hearing loss was present. Hearing loss is known to be a typical symptom accompanying the diabetic phenotype in carriers of the m.3243A>G variant [2]. The phenotypic characteristics of the patients are summarized in Table S5. None of the patients reported any of the seizures or stroke-like episodes typical of this lesion [6].

#### 2.1.2. Other tRNA Variants

**Figure 1:**
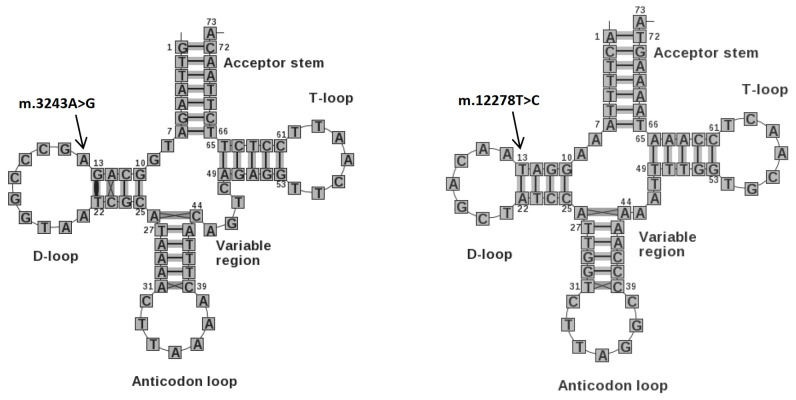
tRNA structures for leucine amino acid with marked variants. (Left: *MT-TL1*; right: *MT-TL2*). Source of images: [8].

The well-known variant described above was accompanied by two more extremely rare variants identified in the tRNA genes for leucine (*MT-TL2*) and arginine (*MT-TR*). Particularly noteworthy is the m.12278T>C variant which is present in the *MT-TL2* gene; this variant has also been recorded in a heteroplasmic form (9%) in a Polish patient with type two diabetes mellitus [7]. This variant is also included in the ClinVar database, and like the m.3243A>G variant it is associated with the MELAS (mitochondrial encephalomyopathy, lactic acidosis and stroke-like episodes) phenotype. The m.3243A>G variant for diabetes also occurs in the tRNA gene for leucine; it is well documented to have damaging effects. Additionally, Figure 1 clearly shows that both variants m.3243A>G and m.12278T>C cause changes at the beginning of the D-loop and may disturb its structure; these alterations can be critical and result in impaired glucose metabolism. Unfortunately, it was not possible to confirm the genotype–phenotype relation in the studied family. Nevertheless, summarizing all the available data, variant m.12278T>C can be considered as pathogenic based on the ACMG classification.

Regarding the overall frequency of these variants, it can clearly be seen that m.3243A>G is much more common in the general population than m.12278T>C (Table 1). In addition, the variant m.12278C>T is not present in a homoplasmic form in the Helix database, unlike m.3243A>G, which was identified in two individuals. This frequency distribution may be due to the m.12278T>C variant having more severe consequences for the functioning of the organism than the m.3243A>G variant; also, like the m.3243A>G variant, it can probably be gradually eliminated from tissues. Unfortunately, it is still unclear whether such elimination occurs, or its rate of occurrence, but such an understanding may be crucial in clinical practice for detecting the m.12278T>C variant and conducting a suitable diagnosis.

No data currently exist on the effect of the m.10426C>T variant in *MT-TR* gene on glucose metabolism. Therefore, more data regarding the phenotype–genotype relationship are needed to confirm this theory. 

### 2.2. rRNA Mutations

We identified four extremely rare variants, two in the gene for 12S rRNA (*MT-RNR1*) and two for 16s rRNA (*MT-RNR2*) (Table 1). Diseases associated with *MT-RNR1* and *MT-RNR2* gene include deafness; aminoglycoside-induced, partial optic atrophy; and nonsyndromic sensorineural, mitochondrial, and auditory neuropathy spectrum disorder [9]. Due to the lack of comparative data, it is difficult to determine whether these variants are causative. To be able to verify their impact, it is necessary to collect data from a larger number of patients with a similar phenotype. 

### 2.3. Variants in Coding Regions

Five extremely rare variants were selected in the coding regions. Three of these were located in the Cytochrome C Oxidase I (*MT-CO1*) gene, and the other two were found in the genes for NADH–ubiquinone oxidoreductase chain 4 (*MT-ND4*) and cytochrome B (*MT-CYB*). Regarding the distribution of the variants, it can be seen that the cytochrome C oxidase gene is over-represented in our study group; indeed, among healthy individuals, the degree of spontaneous variation in the cytochrome C oxidase gene is one of the lowest among all mitochondrial genes [10]. This indirectly indicates that it is probably a key gene for mitochondrial function and thus heavily conserved. 

**Figure 2:**
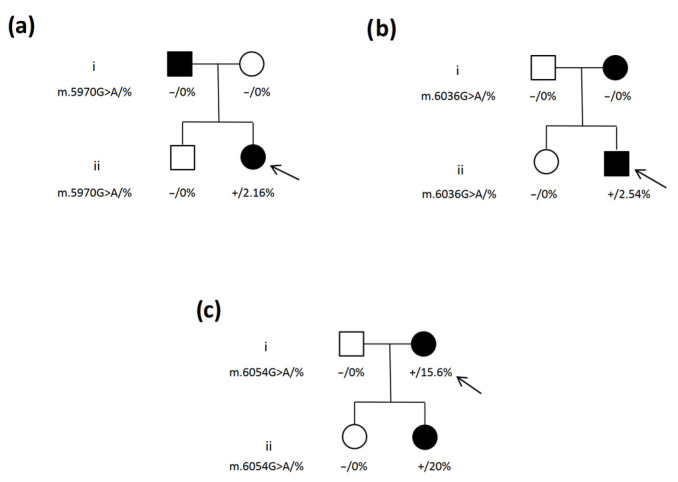
Genetic history of the family with the heteroplasmic variant in the *MT-COI* gene. (**a**) 5970G>A variant, (**b**) 6036G>A variant, (**c**) 6054G>A variant. Genotype is shown underneath each symbol; − and + indicate wild-type and mutant allele, respectively, together with the proportion of heteroplasmic variants in %. The squares represent male family members, and the circles female members. Black-filled symbols denote patients with diabetes; an arrow denotes the proband in the family.

Variants for the *MT-CO1* gene were analyzed in the maternal line in family members (Figure 2). The m.5970G>A variant did not demonstrate any phenotypic linkage and was not found to be present in the genome of relatives, indicating that it may be a de novo mutation (Figure 2a). The m.6036 G>A variant appeared to be inherited in the maternal line, but was not present in the mtDNA of relatives (Figure 2b). This may mean that it is a de novo mutation, or that it may have been selectively removed from the maternal peripheral blood lymphocytes. According to the literature, damaging variants can be selectively removed at a rate of up to 2% per life year [11]; this may be the case considering that the variant is presently at a very low level. However, it is important to note that in both families considered above, children with the MODY phenotype were carriers of *MT-CO1* gene variants.

In the last family, who were found to carry variant m.6054G>A, one of the two children was symptomatic while the other was not. As shown in Figure 2c, correlations can easily be seen between genotype and phenotype: one of the daughters did not carry the variant in the mitochondrial genome and was not burdened with a diabetic phenotype, while the other daughter demonstrated a variant frequency of 20% and an unambiguous clinical phenotype. This analysis suggests that the heteroplasmic variant m.6054G>A in the *MT-CO1* gene may be responsible for the MODY-like phenotype in this family.

However, the fact that one of the daughters has the heteroplasmic variant m.6054G>A and the other lacks it is surprising. This can be explained by the faster selective elimination of damaged mitochondria carrying heteroplasmic variants at the initial stage of embryogenesis. Unfortunately, the exact development of such processes remains unknown, and in some cases the process can take place more quickly than in others [12]. Another likely possibility is that some form of bottleneck effect occurs during the process of inheritance or early division, which leads to the variant being eliminated in one of the family members.

The other two genes where extremely rare variants were found, viz. *MT-ND4* and *MT-CYB*, have never been linked to glucose metabolism and no such reports are available. Again, further studies with larger numbers of patients with a similar genotype are needed to give a definitive answer as to their actual impact on the phenotype.

### 2.4. NUMTs Interpretation Problems

It has long been known that the nuclear genome contains NUMTs of mitochondrial genome insertions of very different lengths [13]. As indicated by many reports, these can pose a problem when interpreting heteroplasmic changes detected by the analysis of short DNA fragments. In some cases, this has led to false conclusions regarding, inter alia, the occurrence of paternal mtDNA inheritance [14]. In our study group, none of the three selected variants were confirmed via long-range PCR, which was most likely due to the presence of NUMTs in the two identified carriers (Table S4). Interestingly, the extremely rare heteroplasmic variant m.3163G>A has also been described in a Polish patient [7]. This indirectly supports that it could be due to the presence of a NUMT because the haplogroups in these individuals are distinct. 

## 3. Materials and Methods

### 3.1. Subjects

All 193 patients who took part in the study had received a diagnosis of hyperglycemia or diabetes mellitus of unspecified etiology according to the WHO definition. BMI (body mass index) was defined in adult patients as body weight divided by the square of body height (kg/m^2^). In children, the BMI value was additionally related to percentiles adjusted for age and sex. In all patients, the following parameters were analyzed: we investigated HbA1c value (%), as an indicator of chronic hyperglycemia, and fasting C-peptide value (ng/mL), as a marker of preserved insulin secretion, in pediatric patients at least two T1DM (type 1 diabetes mellitus) specific antibodies and in adults at least GADA (glutamic acid decarboxylase antibodies) antibodies. The study protocol was approved by the University Bioethics Committee of the Medical University of Lodz, Poland (RNN/41/23/KE).

### 3.2. Library Preparation and Sequencing Reaction

DNA isolation was performed from peripheral blood with a semi-automatic Maxwell RSC Instrument (Promega, Madison, WI, USA). The library was prepared via the Agilent SureSelectQXT Target Enrichment protocol using a custom gene panel with mtDNA enrichment in accordance with the manufacturer’s instructions. Paired-end sequencing was performed in a NextSeq550 System (Illumina, San Diego, CA, USA) at 2 × 150 bp.

The potential contribution of frequent NUMTs insertions for the heteroplasmic variants selected for further analysis was determined via additional sequencing based on long-range PCR. The mitochondrial genome was amplified in one long amplicon (left primer 5′-GGACACTAGGAAAAAACCTTGTAGAGAGAGAGAGAGAG-3′; right primer 5′-AAAGAGAGCTGTTCCTTTGGACTAACA-3′) using Kappa HiFi polymerase (Roche, Basel, Switzerland). The products were then evaluated on an agarose gel. The library was prepared using a Nextera XT DNA Library Preparation Kit (Illumina, San Diego, CA, USA) according to the standard protocol. Paired-end sequencing was performed in a Miseq System (Illumina, San Diego, CA, USA) at 2 × 250 bp. 

### 3.3. Bioinformatic Analysis

Raw FASTQ files were mapped (rCRS, NC_012920.1) using the BWA-MEM alignment algorithm [15] and duplicate reads were removed using the Picard tool [16]. The complete bioinformatic analysis was performed on the local instance of Galaxy [17]. The prepared BAM files were analyzed for contamination, haplogroup, and variants using the local instance of the mtDNAserver algorithm [18]. The potential damaging effect of the indicated variants was evaluated using the following databases: MitoMap [19], MitImpact [20], MitoTip [21], and MtoolBox [22]. Heteroplasmic variants were taken into consideration if minimal coverage was above 300× and if VAF (variant allele frequency) was above 1%. In the case of rRNA gene alterations, the determining criterion of selection was the frequency of the occurrence of the variant in healthy populations. The flowchart of how the variants were selected is presented in Figure S4. Statistical analyses were performed using MedCalc for Windows, (MedCalc Software Version 22.020, Ostend, Belgium).

## 4. Conclusions

Accurate analysis of the mitochondrial genome is a complex process. The literature still lacks an unambiguous interpretation of the variants, and no accurate databases that aggregate information from different sources currently exist. Hence, such an analysis requires a separate methodological approach (the NUMTs problem) and a different pathway for classifying variants than for nuclear variants. The present study identified variants with a low level of heteroplasmy (above 1%), which could be misclassified as very rare variants due to the lack of a well-characterized healthy control group, for example with regard to the occurrence of NUMTs. For variants with such a low level of heteroplasmy, caution should be taken when attempting to determine their pathogenicity, and the results must be confirmed via clear genotype–phenotype correlation in family members or independent reports from other scientific teams. 

The only variants that were pathogenic, or likely pathogenic according to ACMG classification, were m.3243A>G and m.12278T>C. In both cases, the changes occurred in the genes for the amino acid leucine, which indirectly suggests that such alterations in the tRNA are critical and may result in an impaired glucose metabolism. It was also found that the *MT-CO1* gene was over-represented in the study group, suggesting that it may be somehow linked to the MODY-type phenotype. Additionally, a clear phenotype–genotype correlation was observed for the gene variants in one family, suggesting that heteroplasmic variants in *MT-CO1* gene may be responsible for the pathophysiology of glucose metabolism in the human population. However, to definitively verify the impact of the indicated extremely rare variants, it is necessary to examine many mitochondrial genomes with high coverage of a larger group with MODY patients. 

A quarter of a century elapsed from when the first scientific study suggested that the m.3243A>G variant may be pathogenic until the molecular aspect of its effects on mitochondrial and cellular function could be explained [23]. Hence, there is clearly still a lot of work to be conducted if we are to properly characterize the variants of this relatively small genome.

## Figures and Tables

**Table 1:** Selected extremely rare variants in the mtDNA genome from MODY patients.

Patient ID	Gene	Position/ AA Change	Variant Level	Discovery Cohort (%)	Helix Database * (%)	Odds Ratio (95% CI)	*p*-Value	ACMG Classification
19-1225	*MT-RNR1*	811G>A	1	1 (0.5)	10 (0.0051)	100.3 (12.8–787.1)	<0.0001	VUS
19-1643	*MT-RNR1*	1170G>A	0.0545	1 (0.5)	1 (0.00051)	1002.8 (62.5–16,090.3)	<0.0001	VUS
20-737	*MT-RNR2*	2030T>C	0.0863	1 (0.5)	2 (0.00102)	501.4 (45.3–5552.6)	<0.0001	VUS
20-801	*MT-RNR2*	2960T>C	0.0156	1 (0.5)	3 (0.00153)	334.3 (34.6–3227.6)	<0.0001	VUS
19-1777	*MT-TR*	10426C>T	0.0181	1 (0.5)	0 (0)	-		VUS
19-2120	*MT-TL2*	12278T>C	0.0173	1 (0.5)	10 (0.0051)	100.3 (12.8–787.1)	<0.0001	Likely Pathogenic
19-2197, 19-2168, 19-1884, 19-1178,19-1620, 19-1773, 20-1492	*MT-TL1*	3243A>G	0.19;0.444;0.234; 0.0901; 0.2574; 0.2585; 0.3884	7 (3.5)	51 (0.02602)	137.6 (64.7–307.0)	<0.0001	Pathogenic
19-1971	*MT-CO1*	5970G>A; G23S	0.0216	1 (0.5)	3 (0.00153)	334.3 (34.6–3227.6)	<0.0001	VUS
19-1541	*MT-CO1*	6036G>A; G45S	0.0254	1 (0.5)	1 (0.00051)	1002.8 (62.5–16,090.3)	<0.0001	VUS
19-1417	*MT-CO1*	6054G>A; D51N	0.1559	1 (0.5)	9 (0.00453)	111.4 (14.0–883.7)	<0.0001	VUS
20-358	*MT-ND4*	11711G>A; A318T	0.0147	1 (0.5)	1 (0.00051)	1002.8 (62.5–16,090.3)	<0.0001	VUS
20-963	*MT-CYB*	15045G>A; R100Q	0.013	1 (0.5)	1 (0.00051)	1002.8 (62.5–16,090.3)	<0.0001	VUS

* source of discovery cohort [5].

## Data Availability

Data were generated from routine patient diagnostics at the Central Teaching Hospital of the Medical University of Lodz. Data available upon request.

## Supplementary Materials

The following supporting information can be downloaded at: https://www.mdpi.com/article/10.3390/ijms25042438/s1.
