# Supplementary material for: Screening for Rare Mitochondrial Genome Variants Reveals a Potentially Novel Association between MT-CO1 and MT-TL2 Genes and Diabetes Phenotype
Source: Int J Mol Sci. 2024 Feb 19;25(4):2438. doi: 10.3390/ijms25042438 (PMC10889583; doi:10.3390/ijms25042438)
Supplement: Supplementary file 1 [file ijms-25-02438-s001.zip › Figure supplement.pdf]

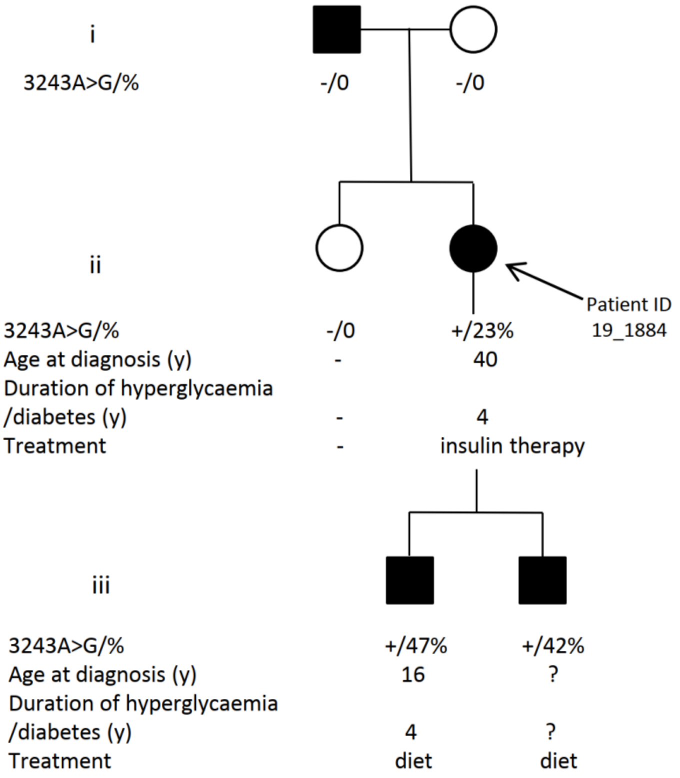

Figure S1. Pedigrees of family (19_1884 patient ID) with heteroplasmic variant 3243A>G. Genotype is shown underneath each symbol; - and +indicate wild-type and mutant alleles, respectively. Below is the genotype, age of diabetes onset in years, duration in years. Squares represent male family members, and circles represent female sex. Black-filled symbols denote patients with diabetes, an arrow denotes the proband in the family.

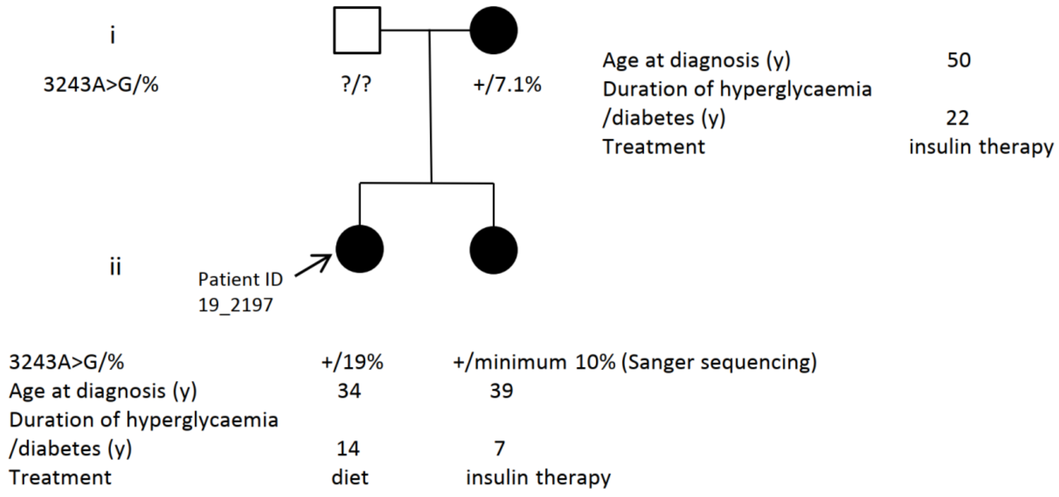

Figure S2. Pedigrees of family (119_2197 patient ID) with heteroplasmic variant 3243A>G. Genotype is shown underneath each symbol; - and +indicate wild-type and mutant alleles, respectively. Below is the genotype, age of diabetes onset in years, duration in years. Squares represent male family members, and circles represent female sex. Black-filled symbols denote patients with diabetes, an arrow denotes the proband in the family.

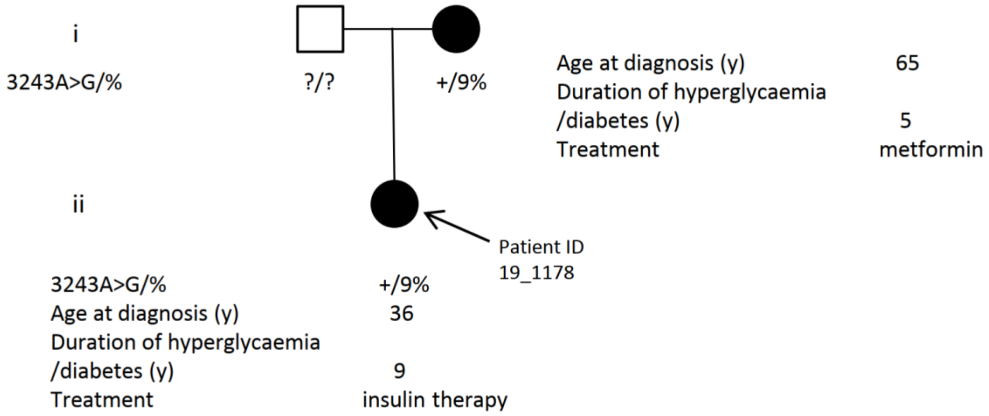

Figure S3. Pedigrees of family (19_1178 patient ID) with heteroplasmic variant 3243A>G. Genotype is shown underneath each symbol; - and +indicate wild-type and mutant alleles, respectively. Below is the genotype, age of diabetes onset in years, duration in years. Squares represent male family members, and circles represent female sex. Black-filled symbols denote patients with diabetes, an arrow denotes the proband in the family.

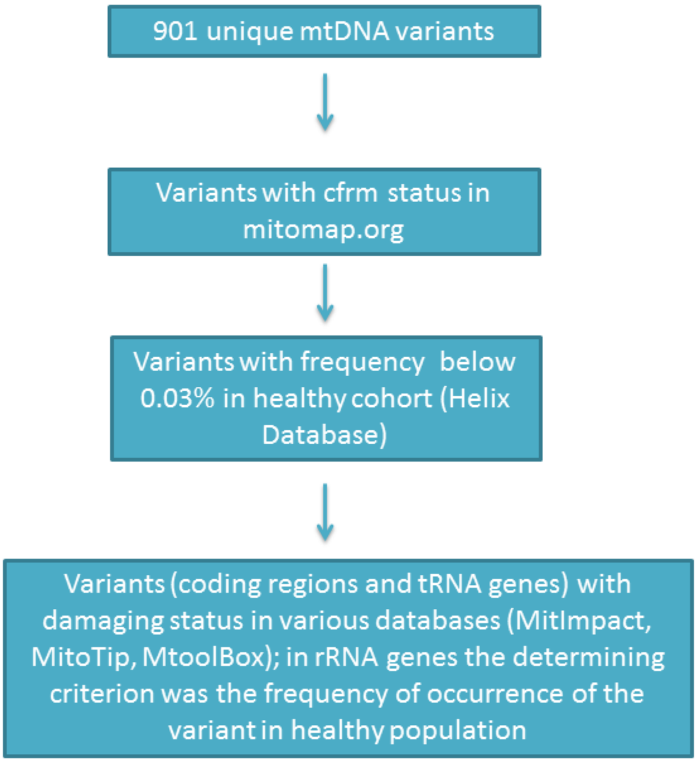

Figure S4. Criteria that were used when selecting variants.

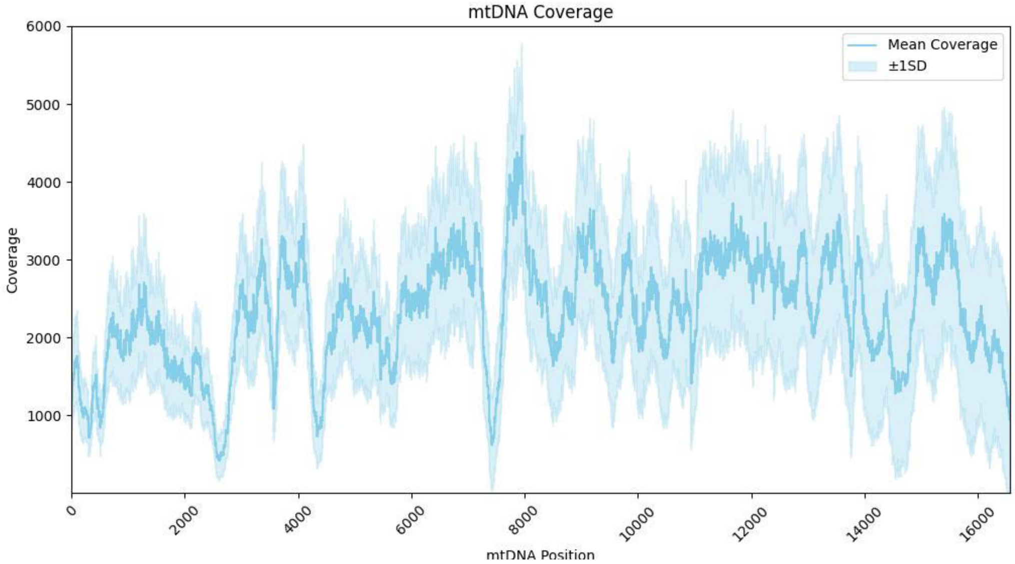

Figure S5. Average coverage across mtDNA genome in all samples.
